# Supplementary material for: Understanding the fine-scale heterogeneity and spatial drivers of malaria transmission in Kenya using model-based geostatistical methods
Source: PLOS Glob Public Health. 2023 Dec 8;3(12):e0002260. doi: 10.1371/journal.pgph.0002260 (PMC10707679; doi:10.1371/journal.pgph.0002260)
Supplement: S1 File — (DOCX) [file pgph.0002260.s001.docx]

# **S1 File**

**Generalized linear Model and generalized linear mixed model description**

Let $Y_{i}$ denote the number individuals that test positive for plasmodium falciparum at survey cluster location $x_{i}$

Then the outcome belongs to the exponential family in this case the binomial family

$Y_{i}$∼*Binomia*l ($m_{i}, P(x_{i})$

Then the GLM model for the probability of a positive test $P(x_{i})$ is expressed as

$$log\left\{ \frac{P(x)}{1-P(x)} \right\}=\alpha+{d\left( x_{i} \right)}^{T}\beta$$

Where $\alpha$ is the intercept parameter and ${d\left( x_{i} \right)}^{T}$ is a vector of observed spatially referenced explanatory variables associated with the response $Y_{i}$, and $\beta$ is a vector of spatial regression coefficients for the covariates.

The generalized liner mixed model introduces the $Z_{i}$ parameter to the Model expressed as :

$log\left\{ \frac{P(x)}{1-P(x)} \right\}=\alpha+{d\left( x_{i} \right)}^{T}\beta$ + $Z_{i}$

Where $Z_{i}$ are mutually independent zero-mean Gaussian random variables
