# Supplementary material for: Understanding the fine-scale heterogeneity and spatial drivers of malaria transmission in Kenya using model-based geostatistical methods
Source: PLOS Glob Public Health. 2023 Dec 8;3(12):e0002260. doi: 10.1371/journal.pgph.0002260 (PMC10707679; doi:10.1371/journal.pgph.0002260)
Supplement: Attachment — Submitted filename: Response to reviewers 4 October 2023.docx [file pgph.0002260.s002.docx]

**Response to reviewers**

***Reviewer 1:***

*First :In the abstract, 'ITN' is used as an abbreviation without its full form explicitly mentioned."*

**Response: the abbreviation has been explicitly mentioned**

*In this context, "spatial drivers" refer to the factors or variables that influence the distribution of malaria cases across geographic space. These influential variables, which play a crucial role in shaping the spatial patterns of malaria risk, will be explicitly outlined in the concluding paragraph of the introduction. Although those that were mentioned subsequently.*

**Response: Thank you for raising this. Due to the word limit restrictions in the abstract section, we have provided the meaning of spatial drivers in the introduction section.**

*in methods section : Even though the IRB and DHS acronyms are generally known, they nonetheless require providing explanations before the abbreviation though mentioned later in another paragraph. It should be mentioned first then should be written abbreviation.*

**Response: We have provided the full meaning of these acronyms.**

The sentence "Malaria Prevalence in five epidemiological zones" could be revised. The term "epidemiological zones" might not be the most commonly used or recognized term in this context. The terminology should be written which are more consistent with standard usage in epidemiological research and might be more appropriate when referring to different areas with distinct epidemiological characteristics.

**Response: We adopted these terms to align with how they are used in the Kenyan context and how they are reported in the Malaria indicator survey. The usage of this term has remained constant in all the surveys that the Kenyan government has reported.**

Fifth: Furthermore, following should be included in the result section as the study focused on Understanding the fine-scale heterogeneity and spatial drivers of malaria transmission using model-based geostatistical methods
1.Geographic Distribution of Malaria Cases: A map displaying the geographical distribution of malaria cases at a fine-scale resolution. This could involve using different colors or shading to represent varying levels of malaria prevalence across different geographic areas. And also mapping of Risk Zones - high-risk or low-risk zones for malaria transmission, these could be delineated on the map.

**Response: The map showing the malaria prevalence at fine scale resolution (5X5 km resolution) is shown in Figure 5.**

2. As the study identifies spatial drivers of malaria transmission, you could overlay environmental factors such as temperature, humidity, and rainfall on the map to show how these factors correlate with the spatial distribution of malaria cases.

**Response: The “correlation” with these environmental covariates is estimated in the binomial geostatistical model output in Table 2. We provide the maps of these factors in Figure 3. It is not possible to show all these on the same map together with the predicted prevalence.**

***Reviewer 2***

Reviewer #2: The paper appears to be the introduction and methods section of a scientific research paper focused on understanding the fine-scale heterogeneity and spatial drivers of malaria transmission in Kenya. The paper aims to use model-based geostatistical methods to analyze malaria prevalence data obtained from the Kenya Malaria Indicator Survey (MIS) and investigate the relationship between malaria transmission and various environmental factors. The research also involves creating high-resolution maps of malaria prevalence to identify hotspots and areas of higher transmission risk. I present my comments in three sections, 1. General comments, 2. Specific comments, 3. Comments on the model. Kindly check and address these comments properly.

General Comments:

1. Clarity and Organization: The introduction provides a comprehensive overview of the malaria situation in Kenya and the significance of the study. Consider breaking down the introduction into smaller sub-sections for better readability.

**Response: thank you for the comment. We ensured that each paragraph addresses a particular theme or idea; this makes it easier to read and follow.**

2. Research Objectives: Clearly state the research objectives and hypotheses in the introduction section. This will help readers understand the purpose of the study more explicitly.

**Response: We have clearly stated the objectives and hypothesis. See lines 100-115**

3. Citations: There are many sentences, facts, and statistics that are not properly cited. Lines 56-85, the researchers used the same reference. Please consider using multiple references. Most of the article paragraphs are referenced to the same citation (#6).

**Response. Thank your for the comment. Reference 6 is the Malaria indicator survey from where we get the cited information on the most recent malaria situation in Kenya. Other likely sources of this information also cite the malaria indicator surveys as their primary source.**

4. Figure and maps: It is not clear why the researchers added all of these maps. I would recommend removing them and use a specific map with clear labels and purpose (check comment #9).

**Response: The maps have different purposes, and it is not possible to combine all these into one. For example, map one aims to show the Kenya Epidemiological Zones while Map 2 shows the locations of the sampled locations in the MIS. This is better presented in separate maps. We have, however, revised our images for better clarity and visualisation.**

5. Methods Explanation: The methods section is detailed and comprehensive. However, consider providing more context on the statistical techniques used, especially for readers who may not be familiar with geostatistical models.

**Response: We have provided the details of this type of method and the associated assumptions in lines 191 to 236. The explanation includes the equations to allow replicability of the methods used.**

6. Variable Definitions: Provide clear definitions of each explanatory variable and its relevance to malaria transmission in Kenya. This will help readers understand the rationale behind the variables chosen for the analysis.

**Response: We have added reference that justify our inclusion of these variables (see line 167).**

7. Data Source and Cleaning: Discuss any potential limitations or biases in the data source and data cleaning process. This will help readers understand the reliability of the dataset used.

**Response: The dataset was clean; we had no missing variables. This was a nationally representative dataset and very reliable.**

8. Ethics and Consent: While you mentioned obtaining permission to use the dataset and ethical clearance, provide a bit more detail on the ethical considerations, participant consent (if applicable), and data anonymization.

**Response: Thank you. The ethical information has been provided separately as part of the journal submission requirements. Briefly Our study involved secondary data analysis using datasets obtained from the Demographic Health Survey (DHS) Program. As mentioned in our previous communication, the original study (The Malaria Indicator Survey) that collected the data had obtained ethical clearance from the Kenyatta National Hospital/University of Nairobi Scientific and Ethics Review Committee.**

**As our study is based on publicly available, de-identified data, we considered seeking additional ethics approval for this particular analysis unnecessary.**

9. Graphical Representation: Use clear, labeled figures and maps that are easy to interpret. Consider providing legends and titles for each figure to enhance their clarity.

**Response: We have revised the figures to include all necessary labels**

Specific Comments:

10. Objectives Clarification: State the specific objectives of the study at the beginning of the "Methods" section. For example, "The objectives of this study were to...".

**Response: This has been added into the revised manuscript**

11. Model Assumptions: Discuss the assumptions underlying the binomial geostatistical model used in the analysis. Highlight any limitations of the model and potential impact on the results.

**Response: We have outlined these in our model description. See line 195-247**

12. Results Interpretation: In the results section, elaborate on the practical implications of the odds ratios and parameter estimates. Explain how changes in the explanatory variables translate to changes in malaria transmission risk.

**Response: These have been outlined in the discussion. See line 310-324**

13. Model Validation: Provide a brief explanation of the empirical variogram technique used for model validation. This will help readers understand the statistical approach used to assess spatial correlation.

**Response: This is discussed in the methods section line232-236**

14. Discussion: In the discussion section, relate your findings to existing literature. Discuss similarities and differences with other studies, particularly those conducted in similar settings.

**Response: We have discussed these see lines 356-362**

15. Policy Implications: Elaborate on the policy implications of your findings. How can the identified hotspots guide targeted interventions and resource allocation in malaria control programs?

**Response: Additional information has been provided in lines 363-365**

16. Study Limitations: Acknowledge any limitations of your study, such as the availability of certain covariates or potential biases in the data. Discuss how these limitations might affect the interpretation of your results.

**Response: We have acknowledged this limitation. Any other unexplained components are included in the model with the variable** $\boldsymbol{Z}_{\boldsymbol{i}}$ **, these are mutually independent zero-mean Gaussian random variables with variance** $\boldsymbol{r}$ **and in this analysis represent the spatial variation within cluster variation, measurement error or small-scale spatial variation. These account for other unmeasured variables and contributes to the model validity.**

17. Future Directions: Suggest potential avenues for future research based on your findings. This could include exploring the effectiveness of specific interventions in the identified hotspots.

**Response: Potential avenue suggestions have been provided in line 368 – 370**

18. Conclusion: Summarize the key findings of the study in the conclusion section, relating them back to the research objectives.

**Response**: **Key findings have been summarized in relation to the study objectives**

Comments on the model:

19. Consider paraphrasing the title of this paragraph: "Model description for the binominal geostatistical Model". You used the word "Model" twice in the title.

**Response: This has been corrected. Thanks**

20. Clarify Covariate Inclusion: In the model description, provide a clear rationale for the selection of covariates used in the binomial geostatistical model. Explain why elevation, ITN usage, mean temperature, rainfall, and urbanization were chosen as predictor variables. Additionally, elaborate on any potential interactions or collinearity among these covariates.

**Response: We have discussed the reasons for inclusion in the line 165- 167. The collinearity of the variables was explored in the exploratory analysis and no collinearity was found.**

21. Matérn Correlation Function Parameters: Provide more detailed explanations for the parameters of the Matérn correlation function used in the spatial component of the model. Explain the significance of the scale parameter () and the smoothness parameter () in the context of malaria transmission. This will help readers understand how these parameters influence spatial correlation and variability.

**Response: Lines 213 to 224 provide the explanations for these parameters in the model**

**Response to comments 22 to 29.**

**We thank the reviewer for this set of comments. We have updated the manuscript pointing the reader to a detailed explanation of model-based geostatistics, that was beyond the objective of this analysis.**

22. Discussion on Variance Parameters: Elaborate on the interpretation and implications of the variance parameters presented in the model. Explain the significance of ² (variance of the Gaussian process), ² (variance of the nugget effect), and their practical implications for the spatial variation of malaria prevalence.

23. Spatial Random Effects: Clarify the concept of spatial random effects (()) and how they capture spatial variation between the sampled clusters. Provide insight into the factors that contribute to the spatial variability, such as geographic features or environmental conditions.

24. Interpretation of Coefficients: In the model formula, explicitly interpret the coefficients associated with each covariate. For example, provide a clear explanation of the interpretation of the coefficient for urbanization (Urban vs Rural) in terms of its effect on the odds of malaria transmission.

**Response: Explanations for the coefficients have been provided in line 285 – 291**

25. Inclusion of Interaction Terms: Discuss whether any interaction terms were considered in the model and if they had a significant impact on the results. Interaction terms could capture complex relationships between covariates and provide a more nuanced understanding of their influence on malaria prevalence.

26. Sensitivity to Parameter Choices: Address the sensitivity of the model results to the choices of parameters such as the Matérn correlation function parameters and variance parameters. Explain whether different parameter choices were tested and their potential impact on the findings.

27. Comparison with Alternative Models: Discuss any alternative modeling approaches that were considered and the reasons for choosing the binomial geostatistical model over other options. Compare the strengths and weaknesses of different modeling strategies, if relevant.

28. Implications of Non-Spatial Variation: Explain the potential implications of the Gaussian random variables () representing small-scale spatial variation, measurement error, or within-cluster variation. Discuss how these sources of variation could affect the overall model results and their interpretation.

29. Model Robustness and Validity: Describe how the robustness of the model was assessed. Discuss any sensitivity analyses conducted to examine the impact of different assumptions or parameter choices on the results. Additionally, elaborate on the methods used to validate the model's assumptions and spatial structure.

**Reviewer 3**

Congratulations to the researchers. This is a well written manuscript with good statistical analysis to identify disease heterogeneity across Kenya and its policy implications.
• Line 234 indicated that there we 98 clusters in lake endemic areas however in the line 237 (table 1) it was 97. Please check and align.

**Response: Thank you . We have made this correction.**

• Line 239: change ‘’figure one’’ to ‘’Figure 1’’ for consistency.

**Response. Thank you. This has been revised.**

• To what extent is the misalignment between spatial and especially paucity of environmental covariates affected the validity of the model's results?

**Response: This was accounted for by the z parameter in the model, the component that accounted for everything else we couldn't explain/ or that was missing. Adding this component made our model valid with the available covariates. Additional evidence of the validity of our model is shown by the variogram presented in the model.**
